# Clinical Outcomes of Abrocitinib in Prurigo Spectrum Disorders: A Case Series

**DOI:** 10.7759/cureus.107619

**Published:** 2026-04-23

**Authors:** Uma Chakravadhanula, Manjula Nagarajan, Shilpa Bhat

**Affiliations:** 1 Dermatology, Institute of Diabetes, Endocrinology, and Adiposity (IDEA) Clinics, Hyderabad, IND; 2 Dermatology, SAAR Clinic, Erode, IND; 3 Dermatology, Subodha Skin, Hair, and Cosmetic Clinic, Bangalore, IND

**Keywords:** abrocitinib, itching, jak inhibitor, prurigo nodularis, prurigo simplex

## Abstract

Prurigo simplex and prurigo nodularis are chronic, pruritic dermatoses with complex, cytokine-driven immunopathogenesis involving the JAK1-STAT signaling pathway. Real-world data on abrocitinib, a selective oral JAK1 inhibitor, in refractory pruritic dermatoses remain limited in the Indian population. Herein, we report four patients with prurigo spectrum disorders who were unresponsive to conventional therapies. Abrocitinib (100-200 mg once daily) resulted in rapid improvement in pruritus, followed by reduction in clinical lesions, including flattening of prurigo-like nodules, decreased pigmentation, and overall improvement in quality of life. No major adverse events were observed. These findings suggest that abrocitinib monotherapy may be an effective and well-tolerated option for refractory prurigo, warranting further evaluation in larger studies.

## Introduction

Prurigo spectrum disorders encompass chronic pruritic dermatoses, including prurigo simplex (PS) and prurigo nodularis (PN), characterized by severe pruritus and symmetrically distributed hyperkeratotic papules or nodules, commonly involving the extensor surfaces of the extremities. Despite standard therapies such as topical corticosteroids, systemic immunosuppressants, and antihistamines, these conditions are often recalcitrant, necessitating exploration of targeted therapeutic options [1].

Abrocitinib, an oral selective Janus kinase-1 (JAK1) inhibitor approved for moderate-to-severe atopic dermatitis, has shown promise in chronic pruritic dermatoses [2]. However, real-world data from the Indian population on its use in prurigo-like disorders remains limited. We report a case series of four patients with refractory prurigo spectrum disorders who demonstrated significant clinical improvement with abrocitinib monotherapy.

## Case presentation

Case 1

**Figure 1:**
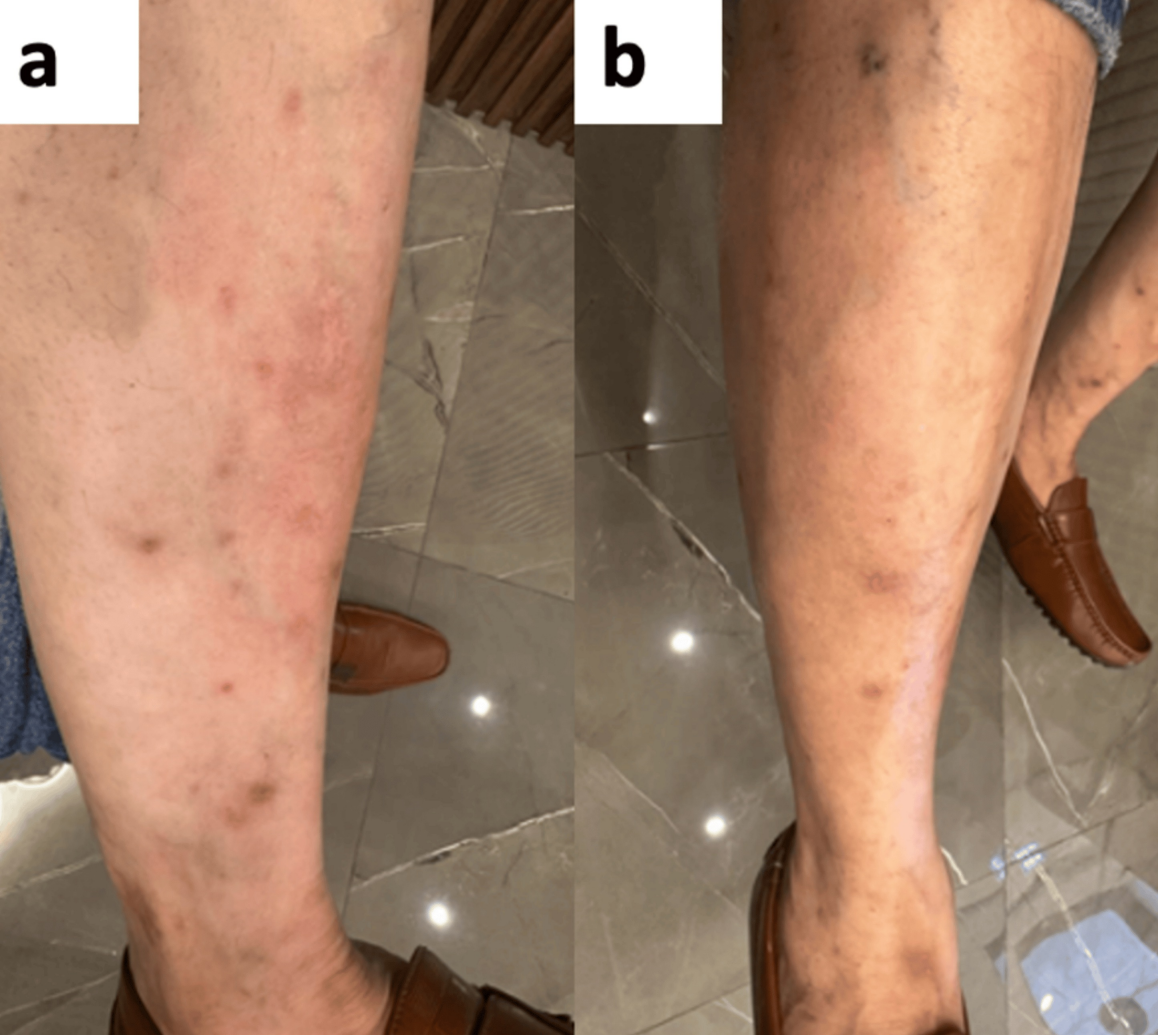
(a) Baseline photograph showing intensely pruritic, hyperpigmented nodules on the leg. (b) At 12 weeks, reduction in nodules and improvement in pigmentation.

A 50-year-old man presented with a 10-year history of persistent, intensely pruritic, hyperpigmented nodules over the forearms and legs, consistent with PS (Figure 1a). The severity of pruritus significantly impaired occupational productivity and quality of life. Previous topical and systemic therapies provided inadequate relief. Given the refractory prurigo, abrocitinib 100 mg once daily was initiated. Within three days, marked improvement in pruritus was reported; by 12 weeks, prurigo-like nodules had flattened, and pigmentation had decreased. Treatment was well tolerated and continued as maintenance therapy at the same dose for three months (Figure 1b).

Case 2

**Figure 2:**
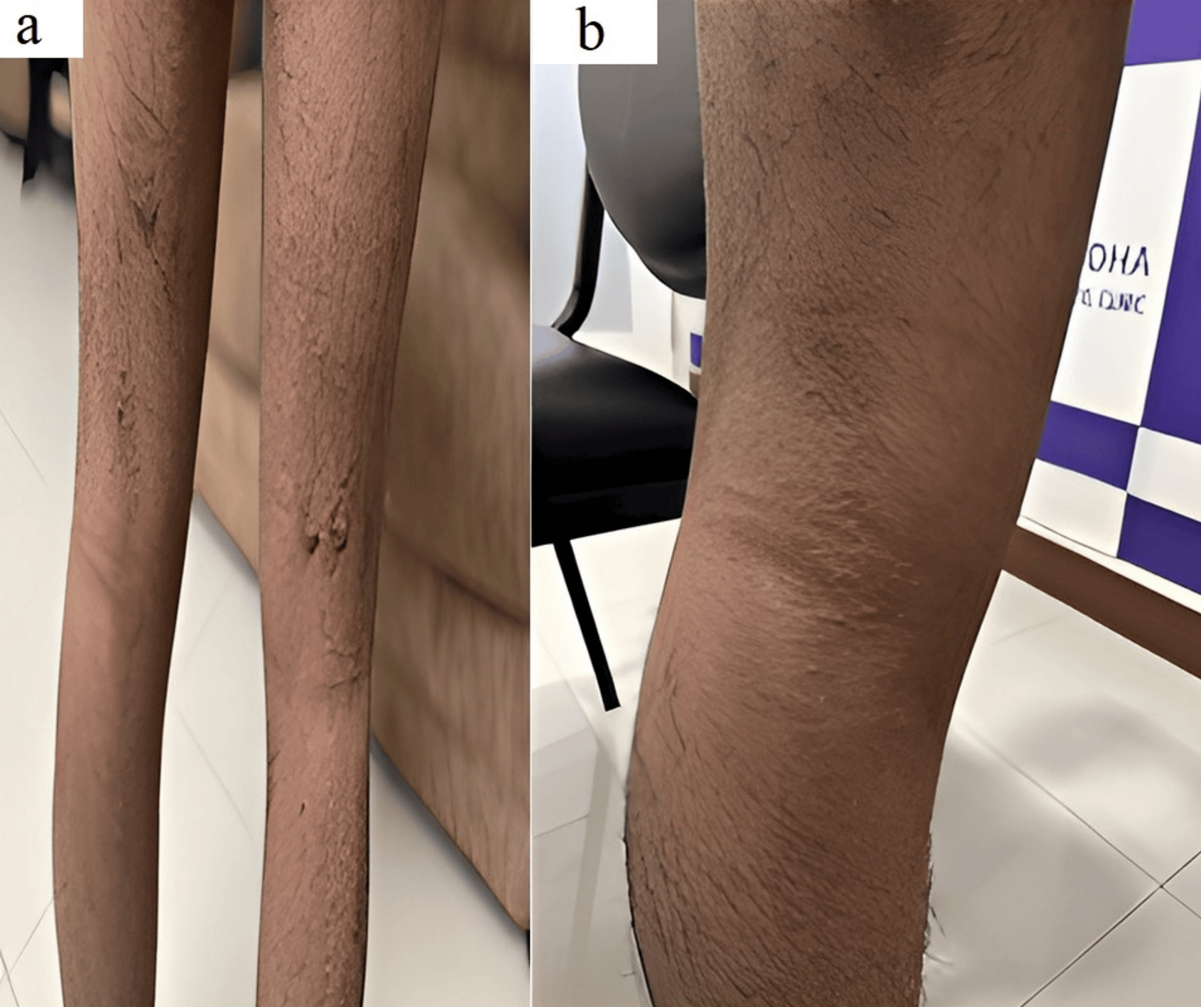
(a) Baseline photograph showing recurrent pruritic nodules on the legs. (b) At eight weeks, reduced pruritus and residual pigmentation.

A 52-year-old woman with type 2 diabetes mellitus and an atopic background presented with recurrent, symmetrical pruritic nodules on the lower limbs for three years, consistent with PN. She had been treated with topical corticosteroids, antihistamines, cyclosporine (200 mg daily), and methotrexate (12.5 mg weekly), with only partial response (Figure 2a). Given refractory prurigo, abrocitinib 100 mg once daily was initiated. Within one week, marked improvement in pruritus and lesion clearance was observed. By eight weeks, prurigo-like nodules had flattened, leaving minimal residual pigmentation (Figure 2b). She was subsequently maintained on pulse therapy for four months with no significant adverse effects.

Case 3

**Figure 3:**
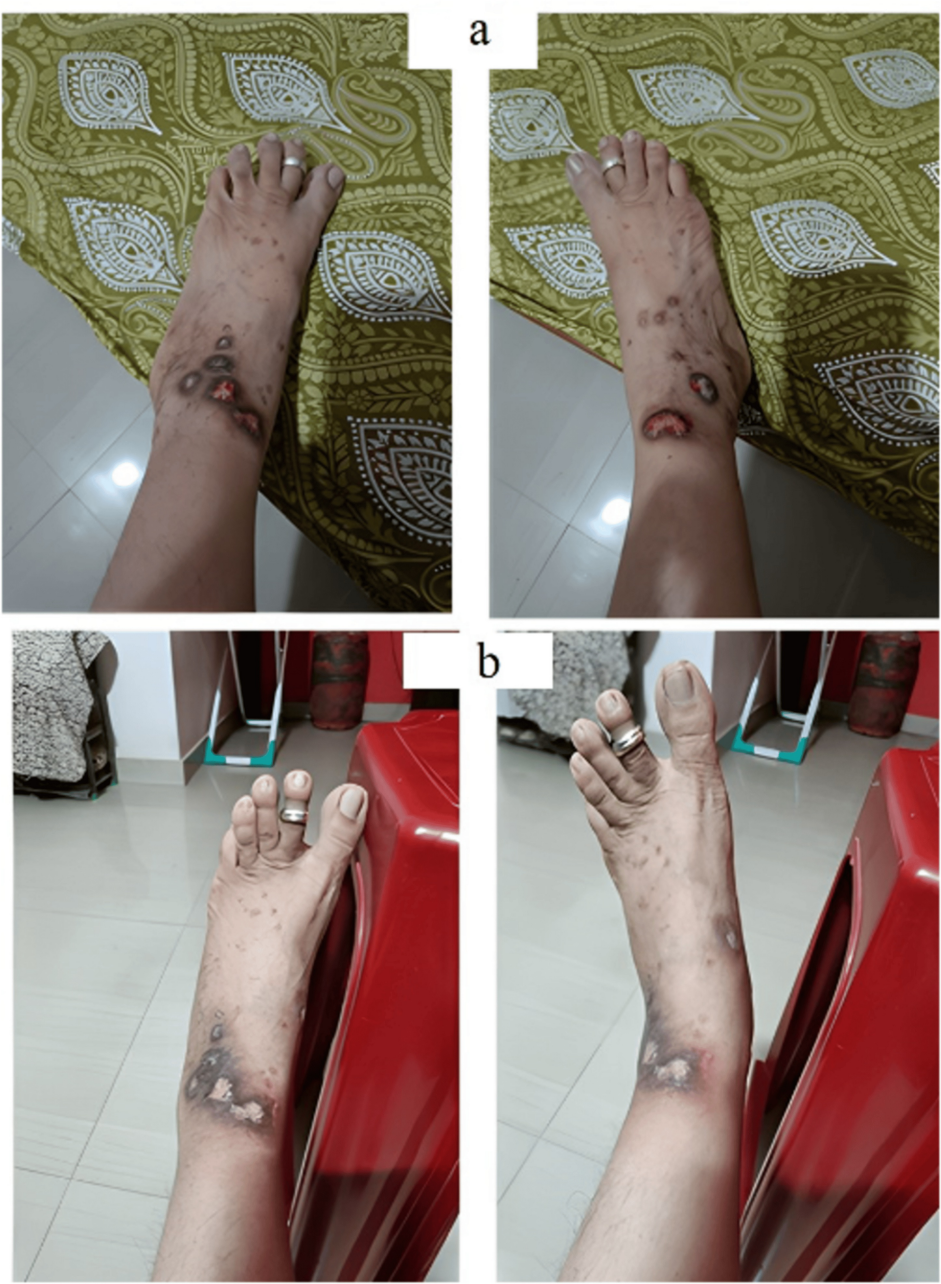
(a) Baseline photograph showing hypertrophic prurigo simplex-like nodules on both lower extremities. (b) After 16 weeks, marked reduction in nodules and flattening with reduced scarring.

An 18-year-old male with childhood-onset moderate eczema presented with widespread erythematous excoriated papules and prurigo-like nodules over the lower limbs (Figure 3a). Previous treatments included cyclosporine and intermittent tofacitinib 5 mg, with an Investigator Global Assessment (IGA) pruritus score of 3. Given inadequate control, abrocitinib 100 mg daily was initiated. Within four days, rapid relief of pruritus was noted. After 16 weeks, prurigo-like nodules had significantly flattened with reduced scarring, and IGA pruritus improved from 3 to 1 (Figure 3b). At the time of writing, the patient remained off systemic therapy and was maintained on emollients without recurrence.

Case 4

**Figure 4:**
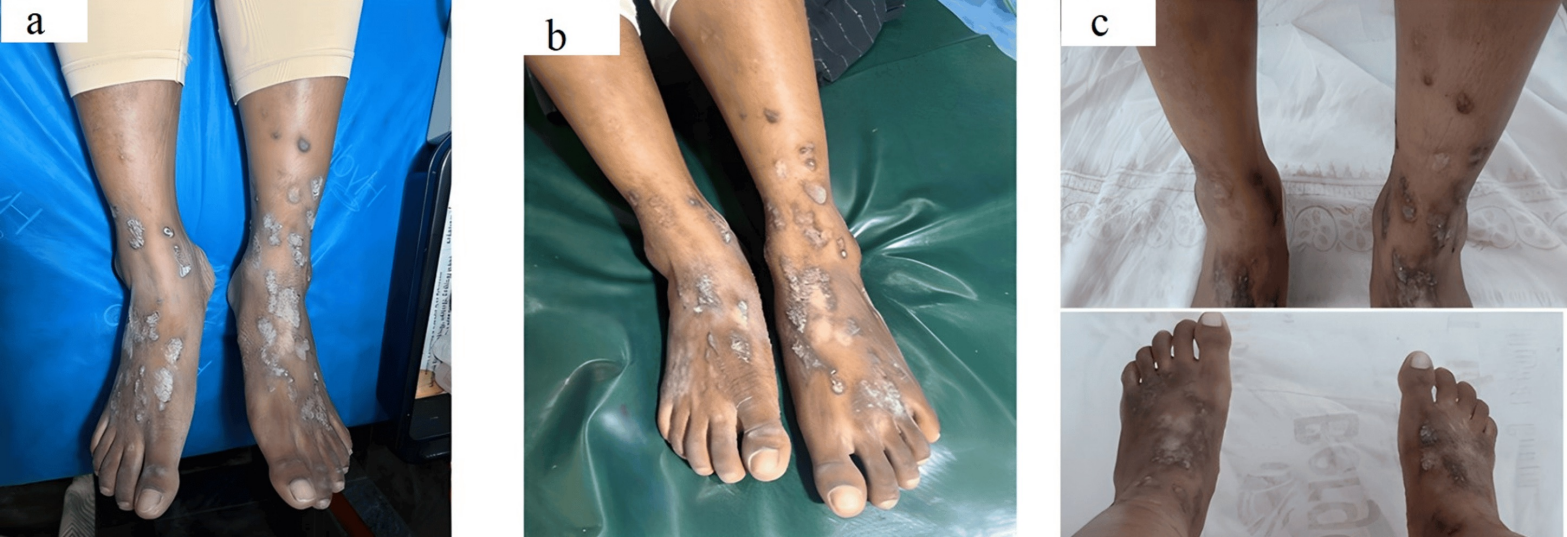
(a) Baseline photograph showing pruritic, lichenified hyperpigmented nodules and plaques. (b) Suboptimal response with 100 mg abrocitinib. (c) Clinical improvement after dose escalation to 200 mg.

A 17-year-old girl presented with severely pruritic, hyperpigmented nodules and plaques over the limbs and dorsal hands and feet for 1.5 years, consistent with PN. Onset followed environmental exposure during home construction, suggesting an irritant trigger. Previous treatments included topical corticosteroids and short courses of antibiotics, with poor control (Figure 4a). Baseline investigations were unremarkable. She was started on abrocitinib 100 mg daily; however, the response was suboptimal at four weeks. The dose was escalated to 200 mg daily (Figure 4b), after which marked improvement in pruritus, function, and quality of life was observed (Figure 4c).

## Discussion

Although the exact pathogenesis of PN remains incompletely understood, current evidence supports a bidirectional interaction between neural and immune pathways contributing to the itch-scratch cycle. Interleukin-31 (IL-31), a key pruritogenic cytokine, has been implicated in multiple chronic pruritic dermatoses and can induce downstream proinflammatory signaling. IL-31 also activates JAK1 and JAK2, key mediators in itch signaling pathways [3].

Given this mechanistic rationale, abrocitinib, a selective JAK1 inhibitor, represents a promising therapeutic option for patients with prurigo spectrum disorders [4]. Emerging case reports and phase II studies have demonstrated its efficacy in prurigo-like disorders, suggesting therapeutic benefit beyond its approved indication in atopic dermatitis [4,5]. While clinical responses to other JAK inhibitors, such as tofacitinib and baricitinib, have been reported in PN, these observations collectively support the role of JAK inhibition in refractory prurigo spectrum disorders.

## Conclusions

This small uncontrolled case series provides preliminary real-world evidence of abrocitinib use in refractory prurigo spectrum disorders, demonstrating rapid and clinically meaningful improvement in pruritus and cutaneous lesions. These findings are exploratory and hypothesis-generating. Prospective controlled studies are required to better define the safety and efficacy of JAK1 inhibition in prurigo spectrum disorders and prurigo-like disorders.

## References

[1] Singh A, Ahmed SS, De A (2025). Use of oral tofacitinib in a case of recalcitrant prurigo nodularis: a promising off-label alternative in resource-poor setup. Indian J Skin Allergy.

[2] Ray A, Pandhi D (2025). Abrocitinib: a comprehensive review of its use in dermatology beyond atopic dermatitis. Indian J Skin Allergy.

[3] Kwatra SG (2020). Breaking the itch-scratch cycle in prurigo nodularis. N Engl J Med.

[4] Sun F, Wu Z (2024). Successful treatment of refractory prurigo nodularis with abrocitinib. Clin Case Rep.

[5] Liang J, Li W, Liu W, Yu Y, Ye H, Zhang X (2024). Abrocitinib monotherapy for refractory prurigo nodularis: report of two successful cases. Clin Cosmet Investig Dermatol.

